# Plasma Proteome Profiling Reveals Inflammation Markers and Tafamidis Effects in V30M Transthyretin Polyneuropathy

**DOI:** 10.3390/ijms26125534

**Published:** 2025-06-10

**Authors:** Karina Nugroho, Chung-yon Lin, Cecilia Monteiro, Teresa Coelho, James J. Moresco, Antonio F. M. Pinto, Evan T. Powers, John R. Yates, Jolene K. Diedrich, Jeffery W. Kelly

**Affiliations:** 1 Department of Chemistry, The Scripps Research Institute, La Jolla, CA 92037, USA; knugroho@scripps.edu (K.N.);; 2 Centro Hospitalar Universitário Santo António, 4050-342 Porto, Portugal; 3 Department of Molecular Medicine, The Scripps Research Institute, La Jolla, CA 92037, USAjyates@scripps.edu (J.R.Y.III); 4 The Skaggs Institute for Chemical Biology, The Scripps Research Institute, La Jolla, CA 92037, USA

**Keywords:** transthyretin familial amyloid polyneuropathy, TTRV30M, tafamidis, plasma, proteomics, immune response, Vyndamax, neurodegeneration

## Abstract

Transthyretin (TTR) variant (V30M) polyneuropathy (ATTRv-PN) is a progressive systemic amyloidosis caused by transthyretin aggregation, leading to a variety of debilitating manifestations, including neuropathy and cardiomyopathy. We investigated the plasma proteome of heterozygotic V30M TTR asymptomatic carriers and heterozygotic V30M ATTRv-PN patients (before and after tafamidis treatment) versus WT TTR healthy control plasma using an organic solvent-induced shift in solubility assay to identify biosignatures for disease progression and therapeutic response. We identified many proteins, including TTR, apolipoproteins, ceruloplasmin, and proteins with functions in innate immunity that displayed changes in either their abundances or their sensitivity to precipitation. Elevated oxidative modifications of TTR and APOE in ATTRv-PN patients suggest a role for oxidative stress in disease pathogenesis/progression. Tafamidis treatment mitigated these pathology-associated changes, suggesting that alleviating proteotoxic stress impacts these other pathways. Although our study was limited to a Portuguese cohort, these findings nevertheless provide a comprehensive plasma proteomic profile of V30M ATTRv-PN patients, V30M TTR carriers, and tafamidis-treated ATTRv-PN patients over up to 60 months; provide insights into ATTRv-PN pathophysiology; identify potential biomarkers for disease progression and therapeutic response; and highlight the utility of proteomics in advancing personalized treatments for amyloidosis.

## 1. Introduction

Transthyretin (TTR) is a tetrameric protein secreted into the blood by the liver that binds and transports holo-retinol binding protein and a small fraction of thyroid hormone under normal physiological conditions. Mutations in the TTR gene can lead to familial transthyretin amyloidosis, which is an autosomal dominant, inherited systemic degenerative disease primarily associated with peripheral neuropathy and/or cardiomyopathy. A valine to methionine substitution at position 30 (V30M) in TTR is one of the most common mutations associated with polyneuropathy [1]. This mutation destabilizes the TTR heterotetramers and homotetramer harboring V30M subunits and promotes their dissociation into monomers, which then misfold and aggregate into structurally heterogeneous aggregates and cross-β-sheet amyloid fibrils that can deposit in peripheral nerves, the heart, and other tissues [2,3]. A typical clinical manifestation of V30M TTR amyloidosis is familial amyloid polyneuropathy (ATTRv-PN), which presents with progressive sensory, motor, and autonomic neuropathy with variable organ involvement and eventually results in incapacitation and death if untreated [4]. Of the 1389 patients with V30M TTR amyloidosis in the THAOS database as of 2021, 64.7% had early-onset disease [5]. The Portuguese patients studied herein generally exhibited early onset (≈30 years old).

Over the last decade, several therapeutics have been regulatory agency-approved for ATTRv-PN treatment, all of which act to decrease the circulating TTR concentration that is vulnerable to dissociation, misfolding, and aggregation. These include (1) small-molecule kinetic stabilizers such as tafamidis [2,6,7] and acoramidis [8,9], wherein kinetic stabilizer binding to TTR prevents TTR tetramer dissociation, thereby preventing aggregation; (2) antisense oligonucleotides such as inotersen and eplontersen [10,11], and (3) RNA interference (RNAi) oligonucleotides such as patisiran and vutrisiran [12,13,14,15], targeting TTR mRNA for degradation predominantly in the liver. These TTR-lowering agents have proven to be successful in reducing circulating TTR levels in plasma by >80%, thereby inhibiting TTR aggregation by reducing TTR to levels below those supporting TTR aggregation and slowing ATTRv-PN. A future potential therapeutic strategy (not yet regulatory agency approved) is gene editing, which is being investigated as a strategy to permanently knock out or knock down hepatocyte-synthesized mutant TTR [16].

Several studies have been published demonstrating the efficacy of tafamidis in halting or slowing the progression of ATTRv-PN [4,17,18,19,20,21] and slowing the progression of variant and age-onset wild-type TTR cardiomyopathy, particularly when initiated early [22]. As with all therapies for TTR amyloidosis, treatment response to tafamidis varies among patients, with approximately one-third of V30M ATTRv-PN treated with tafamidis showing complete cessation of disease progression, one-third exhibiting slowed progression, and one-third not responding [23]. This underscores the need for further studies into a more personalized treatment approach to optimize therapeutic outcomes and enhance the effectiveness of existing therapies. Biosignatures are essential in the management of complex diseases such as ATTRv-PN to enable early detection, more precise diagnosis, and ongoing evaluation of therapeutic efficacy [24,25,26]. In complex degenerative diseases such as ATTRv-PN, early intervention is critical to prevent loss of post-mitotic tissue and clinical debilitation [17,22].

Recently, a model was developed to enhance both ATTRv-PN diagnosis and to predict patient response to tafamidis treatment, based on neurophysiological examination, sex, concentrations of plasma tafamidis and tetrameric TTR [23]. In addition, an immunoassay using an antibody targeting non-native, oligomeric TTR has shown promise for distinguishing tafamidis responders from non-responders [27]. Despite these advances, the underlying pathophysiological mechanism of ATTRv-PN remains incompletely understood. Herein, we utilized differential precipitation of proteins (DiffPOP), proteomics, and peptidomics to comprehensively profile the plasma proteomic differences in heterozygotic V30M ATTRv-PN patients and V30M TTR carriers and the changes in V30M ATTRv-PN patients treated with tafamidis (20 mg once a day) after up to 60 months to identify additional proteome markers of ATTRv-PN and the response of the biomarkers to treatment.

## 2. Results

### 2.1. Plasma Proteome Profiling of Heterozygotic V30M ATTRv-PN Patients and Heterozygotic Asymptomatic V30M Carriers Versus Healthy WT TTR Controls Revealed Changes in Fractionation Profiles of TTR, Apolipoproteins, and Complement Proteins

**Figure 1:**
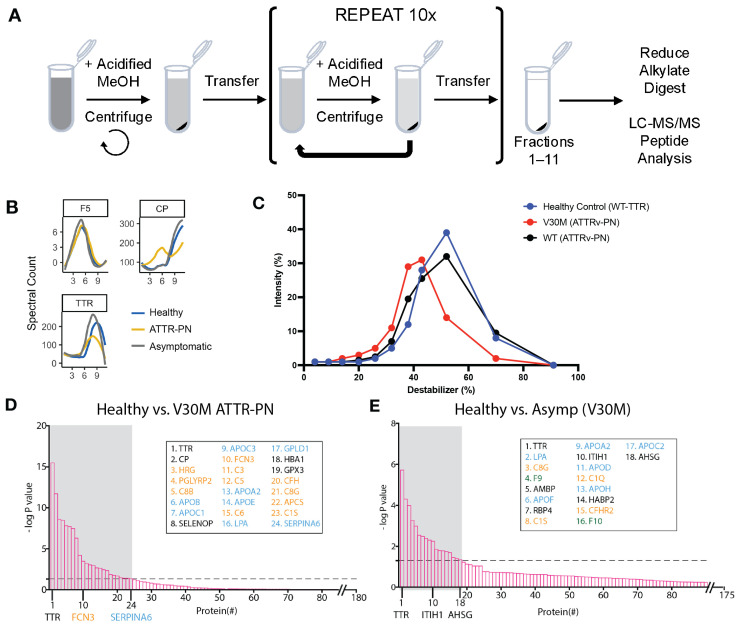
(**A**) Schematic of DiffPOP plasma fractionation workflow. (**B**) Fractionation profile of coagulation factor V (F5), transthyretin (TTR), and ceruloplasmin (CP). (**C**) Fractionation profile of WT TTR peptides in healthy controls, as well as V30M and WT TTR peptides in ATTRv-PN patients. (**D**) All proteins that were identified by TargetSeeker-MS as having significantly different fractionation patterns (24 out of 180) when comparing the plasma proteomes of symptomatic pre-treatment ATTRv-PN patients to healthy controls. Most of the proteins identified were lipid transport (orange) and immune response (blue) proteins. (**E**) All proteins that were identified by TargetSeeker-MS as having significantly different fractionation patterns (18 out of 175) when comparing the plasma proteomes of asymptomatic carriers to healthy controls. Lipid transport (orange) and immune response (blue) proteins are again strongly represented, but coagulation (green) proteins were identified as well when comparing the asymptomatic to healthy samples. The x-axis in (**D**,**E**) represents the rank of the proteins in terms of statistical significance of the differences in fractionation pattern (#), and the y-axis in (**D**,**E**) shows the statistical significance expressed as −log(*p*-value). The dashed line in (**D**,**E**) indicates a multiple-testing-corrected *p*-value = 0.05 as generated by TargetSeeker-MS. The gray boxes in (**D**,**E**) demarcate the proteins that meet this significance threshold.

To establish the proteomic profiles for each experimental group, we analyzed plasma samples from 12 untreated V30M ATTRv-PN patients, 8 asymptomatic V30M carriers, and 4 healthy (WT TTR only) controls; patient samples were chosen at random from our biobank [23]. Plasma was subjected to DiffPOP fractionation by sequential addition of an increasingly concentrated aqueous denaturing acidic methanol solution (Figure 1A).

The integration of DiffPOP methodology with the TargetSeeker-MS algorithm enabled the simultaneous observation of fractionation patterns and relative protein abundance, as quantified by spectral counts. This combined approach provided a comprehensive analysis of fraction distribution and relative concentration across the examined samples. In Figure 1B, we show DiffPOP profiles that exemplify the kinds of differences that are identified by the TargetSeeker-MS algorithm. The profile for coagulation factor V (F5) illustrates the remarkable consistency in DiffPOP profiles that is observed for proteins that do not change as a function of disease state in ATTRv-PN. In contrast, the profile of TTR, the key protein implicated in V30M ATTRv-PN patient proteotoxicity, is illustrative of a protein that changes as a function of disease state primarily in terms of its abundance as opposed to its pattern of fractionation (Figure 1B). Healthy controls and asymptomatic V30M carriers exhibited higher overall TTR spectral counts than V30M ATTRv-PN patients (Figure 1B). This result is consistent with previous observations that TTR plasma concentration decreases as TTR amyloidosis progresses [23,26,28]. Finally, the profile of ceruloplasmin (CP), a copper-containing ferroxidase, is illustrative of a protein that changes as a function of disease state primarily in terms of its pattern of fractionation (Figure 1B). CP precipitates at lower concentrations of acidified methanol from ATTRv-PN patient plasma than from asymptomatic carrier or healthy control plasma (whose CP fractionation patterns are similar to each other). Proteins identified by TargetSeeker-MS on this basis are likely to be physically altered (e.g., are post-translationally modified differently) or to have an altered interactome. Finally, the DiffPOP method is sensitive to the effects of mutations on protein properties, even in the context of heterooligomers. This is illustrated in Figure 1C, wherein we investigated unique V30M peptides in ATTRv-PN patients distinguishable from the corresponding WT peptides. TTR peptides from healthy controls (that only have wild-type TTR) are mostly detected in fractions 8 and 9 (destabilizer concentrations of 43% and 52%) (Figure 1C, blue). WT TTR peptides from V30M heterozygous patients were detected in similar fractions (Figure 1C, black). However, we observed that V30M peptides from the same patient population are more abundant in the earlier fractions 6 and 7 (destabilizer concentrations of 32% and 38%), consistent with our previous findings that TTR heterotetramers become increasingly destabilized as more V30M subunits are incorporated (Figure 1C, red) [29].

Analysis with TargetSeeker-MS identified 24 proteins that have a significantly different DiffPOP profile between the healthy controls and ATTRv-PN patients (Figure 1D). Two main functions associated with this group of proteins are the innate immune response (e.g., the complement proteins C3, C5, and C6) and lipid transport (e.g., the lipid transport proteins APOE and LPA). The fact that we find changes in these pathways between the diseased and healthy states suggests they are either involved in disease pathogenesis or altered as a consequence of the disease process. Using the same methodology, 18 proteins were identified as significantly different between asymptomatic V30M mutation carriers and healthy controls. Proteins involved in innate immunity and lipid transport were again identified as being significantly different in the heterozygotic asymptomatic V30M carriers, showing that these disease-specific proteome signatures appear before symptoms develop (Figure 1E).

### 2.2. V30M ATTRv-PN Disease-Specific Signature of Complement Pathway Activation and Apolipoprotein Alterations

**Figure 2:**
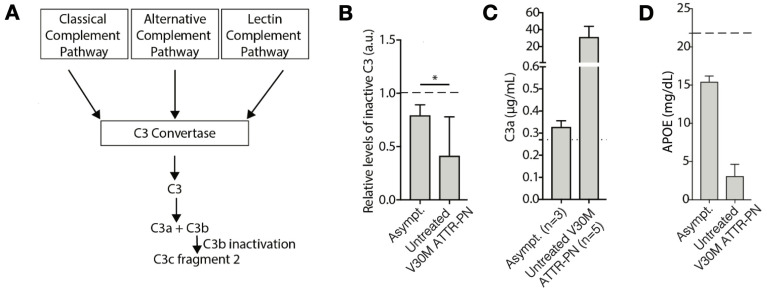
(**A**) Scheme of complement pathways described in this study, which all converge on C3 conversion. (**B**) Quantification of the band corresponding to intact (inactive) C3. The dashed line represents C3 band intensity of the control loaded in each gel. The asterisk (*) indicates significance (*p*-value = 0.05). (**C**) Quantification of plasma C3a levels using a commercially available ELISA. A total of 13/18 untreated V30M ATTRv-PN patients analyzed (not included in the graph) had C3a values above the upper detection limit of the ELISA (>48 µg/mL). The dashed line represents C3a levels in one healthy control. (**D**) Plasma APOE levels by commercially available ELISA in 3 asymptomatic V30M carriers and 18 untreated V30M ATTRv-PN patients. The dashed line represents plasma APOE levels in one healthy control.

**Figure A1:**
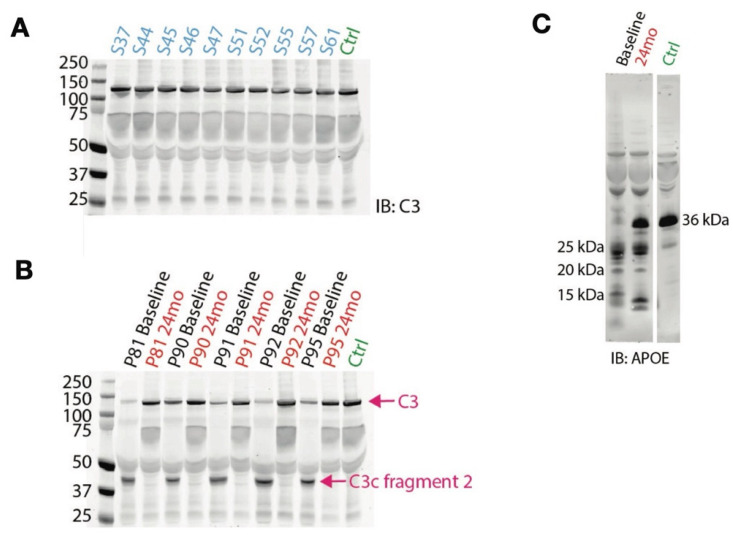
(**A**,**B**) Representative Western blots of whole plasma in SDS-PAGE gel probed with anti-C3 antibody. Lanes labeled with ‘Px’ baseline correspond to untreated V30M ATTRv-PN patients. Lanes labeled with ‘Px 24mo’ indicate V30M ATTRv-PN patients after 24 months of tafamidis treatment. Lanes labeled with ‘Sx’ correspond to asymptomatic carriers. (**C**) Representative Western blots of whole plasma in SDS-PAGE gel probed with anti-APOE antibody. Baseline refers to untreated V30M ATTRv-PN patients, 24mo indicates V30M ATTRv-PN after 24 months of tafamidis treatment, and Ctrl refers to the healthy control.

Many of the proteins that showed significantly different DiffPOP profiles between the healthy controls and both ATTRv-PN patients and asymptomatic V30M carriers were associated with the complement system. Complement variants have been shown to alter the onset of V30M ATTRv-PN [30,31] and are likely to be key components of disease progression. Complement component 3 (C3) plays a central role in complement activation in the three main complement pathways (Figure 2A). In fact, C3 is the most abundant complement protein in plasma, with a normal concentration of ~1.2 mg/mL [32,33]. When complement is active, plasma levels of intact C3 decrease with a corresponding increase in C3 proteolytic cleavage products. Anti-C3 antibodies that recognize the N-terminus of the C3 α-chain can distinguish the 113 kDa inactive form from the proteolytically cleaved active 40 kDa form in a Western blot. We analyzed samples from 3 healthy controls, 20 pre-symptomatic mutation carriers, and 20 ATTRv-PN patients. In the healthy controls, we found that inactive C3 was the predominant form (Figure A1A). An inactive C3 band with a mildly lower intensity (relative to the healthy control cohort) is seen in asymptomatic V30M carriers (Figure 2B and Figure A1B). However, in patients with symptoms, a 40 kDa fragment of C3 is present along with a decrease in the levels of inactive C3 (Figure 2B and Figure A1B). The reduction in inactive C3 concentration likely represents the formation of the C3c fragment 2, which is the N-terminal fragment produced by the inactivation of C3b after C3 has been cleaved (and consequently activated) into C3a and C3b. These data suggest that complement activation starts in asymptomatic V30M carriers, followed by more significant complement activation in symptomatic V30M ATTRv-PN patients. To further scrutinize this hypothesis, we analyzed 18 untreated V30M ATTRv-PN patients using a C3a ELISA-based assay. We found that in 13/18 patients, the levels of C3a were above the linear range of ELISA detection (>48 μg/mL), and in the remaining 5/18 V30M PN patients, the median C3a level was 31.5 μg/mL, an order of magnitude higher than the C3a levels found in the V30M asymptomatic carriers (Figure 2C).

Changes in the DiffPOP precipitation pattern of different apolipoproteins is the other main component of the V30M ATTRv-PN-specific signature shown here. To explore this finding, we further examined Apolipoprotein E (APOE). As noted above, the DiffPOP fractionation pattern of APOE was significantly different between V30M ATTRv-PN patients and healthy controls, suggesting differences in post-translational modifications or processing (Figure 1D). Being homozygotic for the APOE4 isoform is the most profound risk factor for developing Alzheimer’s disease (AD) [34]. Levels of APOE in the plasma of patients with AD correlate with levels of Abeta 1-42 [35]. To test whether this was also seen in the plasma of patients with V30M ATTRv-PN, we quantified plasma levels of APOE using an ELISA assay in 3 asymptomatic V30M carriers and 18 untreated V30M ATTRv-PN patients. We found that APOE concentration in plasma is low in untreated V30M ATTRv-PN patients and higher in asymptomatic V30M carriers (Figure 2D).

### 2.3. Elevated Oxidative Modifications

**Figure 3:**
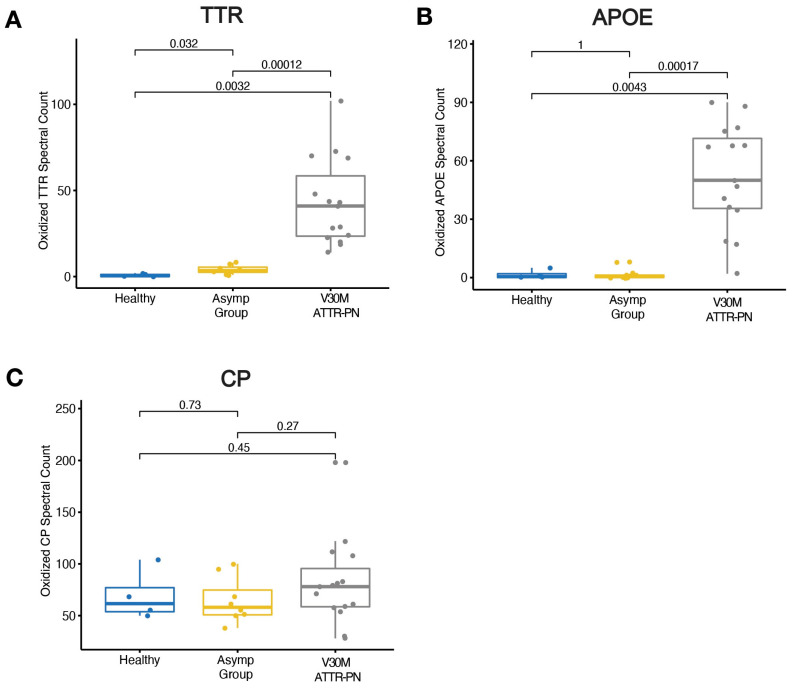
(**A**) Spectral counts of oxidized TTR in the healthy control, asymptomatic V30M carriers, and V30M ATTRv-PN patient plasma. (**B**) Spectral counts of oxidized APOE in the healthy control, asymptomatic V30M carriers, and V30M ATTRv-PN patient plasma. (**C**) Spectral counts of oxidized CP in the healthy control, asymptomatic V30M carriers, and V30M ATTRv-PN patient plasma.

The changes in the DiffPOP precipitation profiles of TTR, CP, and APOE suggested that these proteins were in an altered state in V30M ATTRv-PN patients and/or asymptomatic V30M carriers versus healthy controls. One mechanism by which these changes in precipitation profiles could occur is by oxidative stress-associated protein post-translational modifications, which are known to play various roles in pathophysiological processes [36,37]. We conducted a comprehensive analysis of our DiffPOP proteomics dataset, focusing on oxidative modifications of cysteine to cysteic acid, methionine to methionine sulfoxide and/or methionine sulfone, and carboxymethylation of lysine [38]. We found that V30M ATTRv-PN patients had a significantly higher level of oxidized TTR (*p* < 0.01) (Figure 3A) and APOE (*p* < 0.01) (Figure 3B) spectral counts relative to the healthy control and asymptomatic V30M cohorts. Interestingly, no significant differences were observed in oxidized CP spectral counts across the groups (Figure 3C). These findings underscore the prevalence of oxidative modifications in the plasma proteome of V30M ATTRv-PN patients; however, these modifications may not be uniform, instead targeting only a subset of the plasma proteome. Nevertheless, these results strongly suggest a prominent role for oxidative stress in ATTRv-PN.

### 2.4. Peptidomics Analysis Reveals Aberrant Proteolytic Activity and a Signature of Inflammation

It has been reported that V30M ATTRv-PN patients have abnormally high endogenous protease activities in their plasma [39]. The peptides that would result from such activity would likely remain in the soluble fraction throughout the DiffPOP fractionation procedure. Therefore, we used this as a proxy to determine if our cohort of V30M ATTRv-PN patients had elevated protease activity in their plasma. We evaluated the peptidome (peptides that remain soluble after DiffPOP fractionation) from 8 healthy controls, 43 asymptomatic V30M carriers, and 45 untreated individuals diagnosed with V30M ATTRv-PN.

**Figure 4:**
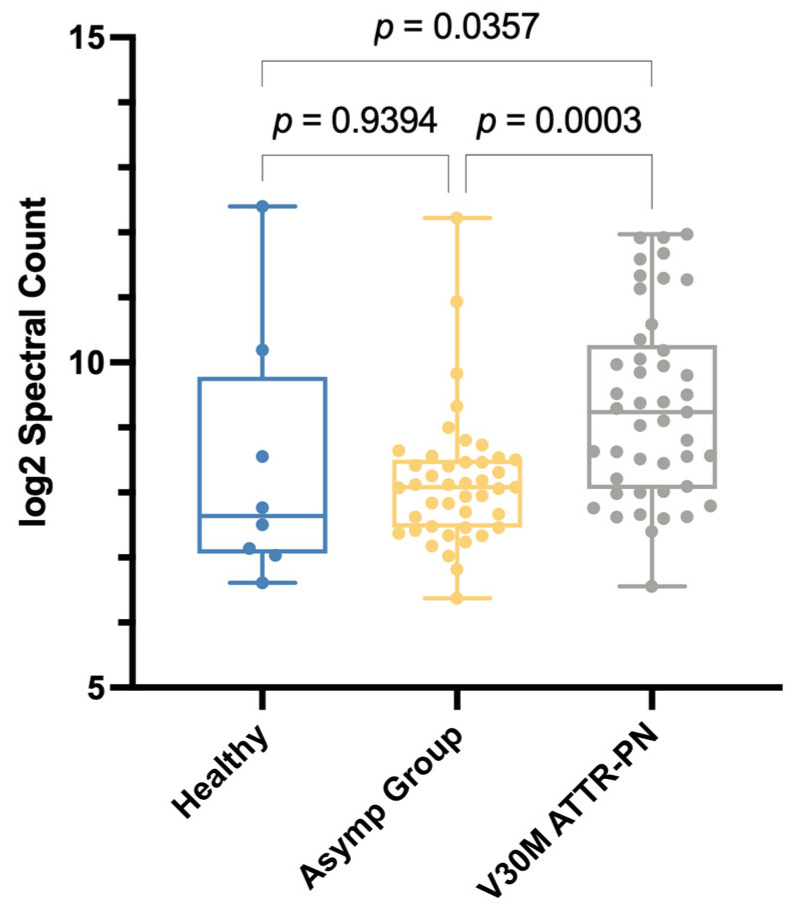
Spectral counts of peptides that remain soluble after DiffPOP fractionation in healthy control plasma (*n* = 8), asymptomatic V30M carrier plasma (*n* = 43), and untreated V30M ATTRv-PN patient plasma (*n* = 45). Statistical analysis was performed using the Kruskal–Wallis test with the Benjamini–Hochberg correction.

We found that the V30M ATTRv-PN patient samples had significantly higher total spectral counts when compared to asymptomatic V30M carriers (*p* = 0.0003) and the healthy controls (*p* = 0.0357) at baseline (Figure 4). The elevated spectral count is consistent with elevated protease activity. The asymptomatic V30M carriers had spectral counts comparable to the healthy controls.

**Figure A2:**
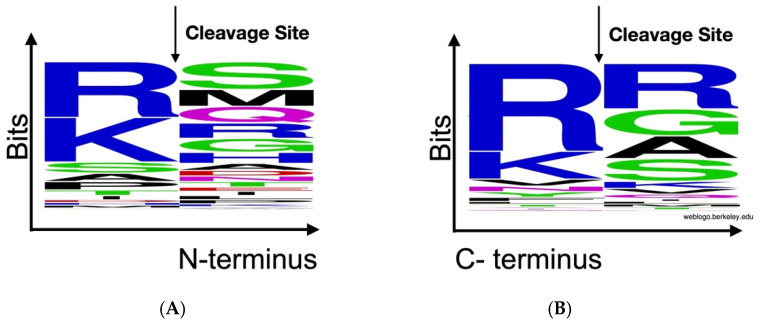
Residue frequencies at cleavage sites of proteolyzed peptides from our peptidomics dataset at (**A**) fragment N-terminus and (**B**) fragment C-terminus.

To investigate the nature of this protease activity, we determined the amino acid frequencies at the N- and C-termini of the cleavage sites in the proteins from which the peptides were derived. This revealed that cleavage most often occurred after arginine and lysine residues, indicative of trypsin-like serine proteases (Figure A2A,B).

### 2.5. Effects of Tafamidis Treatment on Proteolysis and Oxidative Modification in the Proteome

Tafamidis treatment slows or stops the progression of V30M ATTRv-PN in 70% of treated patients [23]. To determine whether the plasma proteomic changes we have observed in V30M ATTRv-PN patients were normalized by kinetic stabilizer therapy, we applied the DiffPOP technique to plasma samples of V30M ATTRv-PN patients in our cohort after 24 months of tafamidis treatment. The previously described 45 V30M ATTRv-PN patients who received tafamidis for 24 months were further categorized into non-responder and responder status as described previously [23].

**Figure 5:**
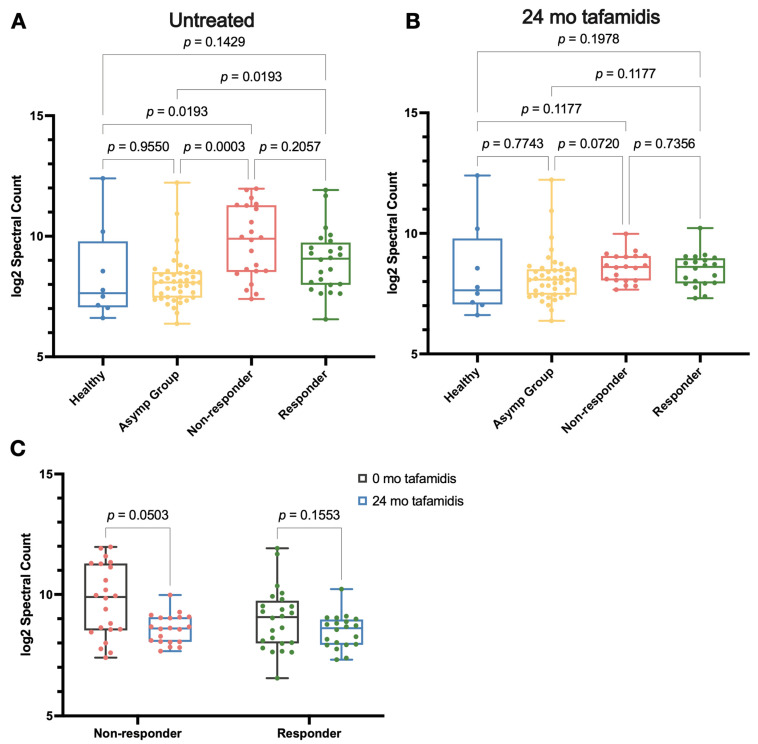
(**A**) Spectral counts of peptides that remain soluble after DiffPOP fractionation in the healthy control (*n* = 8), asymptomatic V30M carriers (*n* = 43), and untreated V30M ATTRv-PN patient plasma (non-responder: *n* = 22 and responder: *n* = 24). Statistical analysis was performed using the Kruskal–Wallis test with the Benjamini–Hochberg correction. (**B**) Spectral counts in the healthy control (*n* = 8), asymptomatic V30M carriers (*n* = 43), and V30M ATTRv-PN patient plasma (non-responder: *n* = 20 and responder: *n* = 20) following 24 months of tafamidis treatment. Note that the data for the “Healthy” and “Asymp Group” are the same in panels (**A**,**B**). (**C**) Comparison of spectral counts in V30M ATTRv-PN patient plasma from panel (**A**,**B**) before (non-responder: *n* = 22 and responder: *n* = 24) and after 24 months of treatment with tafamidis (non-responder: *n* = 20 and responder: *n* = 20). Note that the data in panel (**C**) are replotted from panels (**A**,**B**) for easier comparison. Statistical analysis was performed using the Mann–Whitney test with the Benjamini–Hochberg correction.

We first investigated whether tafamidis could rectify aberrant proteolysis in V30M ATTRv-PN patients by measuring spectral counts. Herein, we compared V30M ATTRv-PN patients treated with tafamidis after 24 months, asymptomatic V30M carriers, and healthy controls. As noted above, the V30M ATTRv-PN patient group exhibited statistically elevated spectral counts compared to the healthy control (*p* = 0.0357) (Figure 4). Before tafamidis treatment, the non-responder group showed significantly elevated spectral count (*p* = 0.0193), while responders had modestly elevated spectral count (*p* = 0.1429) compared to healthy controls (Figure 5A). In V30M ATTRv-PN patients who received 24 months of tafamidis treatment, the spectral count was decreased to levels comparable to healthy controls (Figure 5B). In both non-responder and responder groups, spectral counts had decreased after 24 months of tafamidis treatment compared to the baseline at t_0_, although this trend did not reach statistical significance (*p* = 0.0503 and *p* = 0.1553, respectively) (Figure 5C).

**Figure A3:**
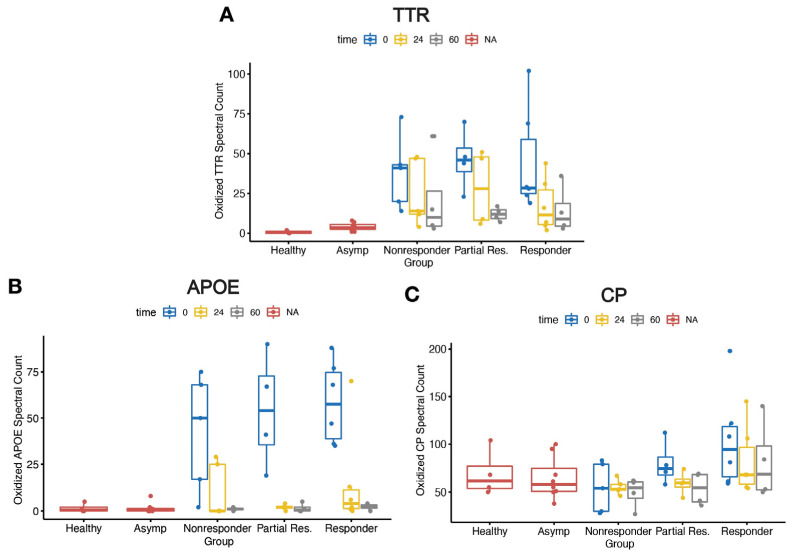
(**A**) Spectral count of oxidized TTR in healthy control (*n* = 4), asymptomatic (*n* = 8), and V30M ATTRv-PN patient plasma at 0, 24, and 60 months after tafamidis treatment. The V30M ATTRv-PN patient cohort is classified into non-responder, partial responder, and responder. At baseline (t_0_), the sample sizes were as follows: non-responders (*n* = 4), partial responders (*n* = 4), and responders (*n* = 6). At the 24-month timepoint (t_24_), the sample sizes were as follows: non-responders (*n* = 5), partial responders (*n* = 4), and responders (*n* = 6). At the 60-month timepoint (t_60_), the sample sizes were as follows: non-responders (*n* = 4), partial responders (*n* = 4), and responders (*n* = 4). (**B**) Spectral count of oxidized APOE in healthy control, asymptomatic, and V30M ATTRv-PN patient plasma at 0, 24, and 60 months after tafamidis treatment. (**C**) Spectral count of oxidized CP in healthy control, asymptomatic, and V30M ATTRv-PN patient plasma at 0, 24, and 60 months after tafamidis treatment. Due to the limited sample size for each response category, statistical analyses were not performed to prevent overinterpretation of these data. We therefore only note trends in the data.

We then investigated the level of oxidative modification in TTR and APOE, which were shown above to be extensively oxidatively modified in untreated V30M ATTRv-PN patients. We investigated 15 V30M ATTRv-PN patients in our cohort after 24 months of tafamidis treatment, consisting of 6 responders, 4 partial responders, and 5 non-responders. We found that oxidative modification of TTR trended downwards after 24 months of treatment and was lowered after 60 months of treatment (Figure A3A). Additionally, oxidative modification in APOE decreased after 24 months of tafamidis treatment and was comparable to healthy controls after 60 months of treatment in all patient subgroups (responders, partial responders, and non-responders) (Figure A3B). Interestingly, although oxidative modification in CP trended lower, the differences observed in CP spectral counts across the groups were not statistically significant (Figure A3C). These findings suggest that tafamidis seems to generally alleviate oxidative stress.

## 3. Discussion

### 3.1. DiffPOP Is an Effective Method for Clinical Biomarker Discovery

Changes in the plasma proteome are often signs of disease onset. Sometimes these are obvious (for example, increases in alanine aminotransferase (ALT), indicating liver injury) [40], and sometimes they are more subtle (for example, recently described changes in plasma Abeta 1–40 and 1–42 and phospho-tau indicating the onset of Alzheimer’s disease) [41,42,43], but either way these protein changes can be helpful for disease diagnosis. Finding novel biomarkers in the plasma proteome is particularly challenging because the most abundant proteins (especially albumin) often hinder the detection of less abundant proteins [44]. In this report, we approached the challenge with a solubility-based protein separation method, DiffPOP [45]. Even though DiffPOP was previously used for identifying drug-target interactions by observing changes in protein precipitation behavior upon drug binding [45,46], we envisioned that adapting this non-biased fractionation method to plasma proteomic analysis would not only add an additional dimension to the analysis—that is, a protein’s resistance to precipitation as well as its abundance—but also lessen the aforementioned analytical burden. This method enabled us to build a comprehensive proteomic profile of the plasma proteomes of healthy controls, asymptomatic heterozygotic V30M carriers, and symptomatic V30M ATTRv-PN subjects. We believe that the DiffPOP method described herein will be broadly applicable to the analysis of many types of biological samples, from plasma to cerebrospinal fluid to cell homogenates.

### 3.2. Inflammation Is a Key Feature of ATTRv-PN Pathological Progression

Several aspects of our data suggest that inflammation is active in untreated ATTRv-PN patients. The most direct evidence is the depletion of C3 in the ATTRv-PN patients, likely by its proteolytic cleavage (Figure 2), which also manifests in the elevated levels of proteolysis-derived peptides in patient plasma compared to healthy controls (Figure 4), strongly suggesting inflammation. Moreover, the increased extent of oxidative modification of plasma proteins (Figure 3), suggestive of oxidative stress, is also consistent with inflammation. Finally, changes in the levels of apolipoproteins, which are observed in ATTRv-PN patients (Figure 1D,E), are also suggestive of inflammation [47].

Our evidence also suggests that inflammation builds as ATTRv-PN progresses. All the signs of inflammation cited above are present in patients that are symptomatic for the disease, but many are also present to a lesser extent in asymptomatic V30M mutation carriers, especially C3 conversion (Figure 2B,C) and changes in apolipoproteins (Figure 1E and Figure 2D). Complement protein regulation and inflammation alterations in transthyretin amyloidosis progression have been previously reported [48,49], and our observation adds to the intriguing possibility that markers of inflammation could be used to inform decisions about when to start treating patients with tafamidis or another disease-modifying ATTR therapy.

It is interesting that CP is one of the plasma proteins whose fractionation profile changes the most in ATTRv-PN patients vs. healthy controls. CP functions as the major copper transport protein in plasma and as a ferroxidase, which reduces reactive oxygen species (ROS) by oxidizing Fe^2+^ to Fe^3+^ in cells and releasing it to apotransferrin [50]. Thus, CP plays a critical role in iron homeostasis and oxidative stress regulation. The changes in CP may reflect the body’s response to higher oxidative stress and could be disruptive in these pathways [36,50,51,52,53,54].

Similarly, the distinct APOE fractionation patterns across all three experimental cohorts may be indicative of systemic effects, given the role that APOE plays in other neurodegenerative diseases as well as regulation of immune processes and the established role of lipid dysregulation in neurodegeneration [55,56,57]. The APOE gene is one of the strongest risk factors for sporadic Alzheimer’s disease, and its polymorphisms impact critical aspects of AD pathology, including neuroinflammation and amyloid-β load [58]. APOE is also central to lipid metabolism in the CNS and mediates the binding of lipoproteins to cell-surface receptors [56,59]. Lipid metabolism is highly dependent on reactive oxygen species, which may be exacerbated and out of balance due to the altered redox environment in V30M ATTRv-PN patients described above [60,61]. These findings collectively highlight the broader proteomic disruptions that accompany V30M ATTRv-PN progression and further implicate inflammation, oxidative stress, and lipid dysregulation as key drivers of V30M ATTRv-PN pathogenesis.

### 3.3. Proteome Regulation by Tafamidis

The findings from our study provide compelling therapeutic evidence that tafamidis treatment addresses some of the processes described above. For example, in the five patient samples studied, C3 conversion is greatly reduced upon treatment with tafamidis for 24 months (Figure A1B), suggesting mitigation of inflammation. In that same subset of patients, tafamidis also demonstrated a progressive reduction in oxidative modifications of TTR over the 24- and 60-month treatment periods (Figure A3B), as well as APOE (Figure A3C). Also, our analysis of proteolytic activity revealed that both asymptomatic V30M carriers and pre-treatment V30M ATTRv-PN patient cohorts in this study exhibited elevated spectral counts at baseline, indicating increased proteolysis. After 24 months of tafamidis treatment, V30M ATTRv-PN patients showed a significant decrease in peptide levels (Figure 5). This result suggests that tafamidis helps normalize aberrant proteolytic processes that are dysregulated in V30M ATTRv-PN, likely due to inflammation.

The progressive improvement seen over 24 and 60 months emphasizes the importance of long-term treatment in managing V30M ATTRv-PN. Moreover, the differential response among patient subgroups highlights the importance of utilizing biomarkers to guide treatment decisions. The observed changes in TTR and APOE oxidation and overall proteolysis suggest that tafamidis may have more extensive effects on protein homeostasis than previously described.

## 4. Materials and Methods

### 4.1. Study Design

This study was a non-randomized, longitudinal observational study of ATTRv-PN patients that started tafamidis therapy in a single clinical center (Unidade Corino de Andrade) with at least 24 months of follow-up from baseline. The dose and treatment interval of 20 mg once a day were determined by using a pharmacokinetic/pharmacodynamic model to achieve serum tafamidis/TTR ratios of approximately 2:1, as described in the original clinical trial for tafamidis [2]. Tafamidis therapy eligibility was determined based on the following criteria: 1. confirmed TTR V30M mutation; 2. Congo red positive amyloid deposition on the tissue biopsy; and 3. symptoms confirming the involvement of the peripheral nervous system. Exclusion criteria were the following: 1. patients in clinical trials Fx005 (NCT00409175) and Fx006 (NCT00791492); 2. patient inclusion in the patisiran trial (NCT01961921); 3. comorbidity of other central and peripheral nervous disorders; 4. therapy suspension before 60 months; 5. loss to clinical follow-up; 6. lack of plasma/serum samples; and 7. refusal to participate in this study. The original patient cohort had 306 patients, from which 96 were excluded for various reasons described in our previous publication [23]. Only one patient died out of the 306; they died of an unknown cause and hence were excluded due to the inability to classify their response. The study was approved by the ethical and institutional review boards at the Centro Hospitalar do Porto and The Scripps Research Institute prior to subject enrollment. Additionally, plasma samples of V30M ATTRv-PN carriers were also obtained from the same clinical center, Unidade Corino de Andrade. Plasma from the healthy controls was obtained from the Scripps Research Institute Normal Blood Donor Services Center. Each subject provided written informed consent.

### 4.2. Differential Precipitation of Proteins (DiffPOP)

We performed proteomic profiling to investigate proteome signatures in three patient populations: (1) a diagnosed V30M ATTRv-PN polyneuropathy patient population, (2) asymptomatic V30M ATTRv-PN carriers, and (3) healthy controls; and to investigate whether proteomic changes occur in diagnosed V30M ATTRv-PN patients who received treatments. Plasma proteomic studies are notoriously complex due to the dynamic range and high abundance of a few proteins, such as albumin and immunoglobulins, which can mask the detection of less abundant but potentially disease-relevant proteins. In humans, albumin accounts for approximately half of the protein in plasma, creating significant challenges for plasma proteomics analysis [28].

To address this challenge, we adopted a sample fractionation method that has been developed recently called DiffPOP (Differential Precipitation of Proteins) that utilizes increasing acidified methanol concentrations to selectively precipitate proteins. DiffPOP highlights solubility differences between proteoforms and offers valuable insights that are often obscured in traditional bottom-up proteomics approaches [29]. To perform the DiffPOP fractionation, we adopted a previously published protocol [29]. In brief, 7.5 µL of plasma was diluted in 142.5 µL of water and 100 µL of Buffer A (Clontech, San Jose, CA, USA, Cat. #635626). The DiffPOP method was carried out with sequential additions of the denaturing solution of 90% methanol/1% acetic acid (10, 15, 17, 20, 24, 30, 35, 40, 80, 300, and 2000 µL). Each addition was followed by vigorous vortexing and centrifuging (18,000× *g* for 10 min at 4 °C). The supernatant was transferred to a new Eppendorf tube, more denaturing solution was added to it, and the sample was vortexed and centrifuged. The process was repeated to produce eleven pelleted fractions. All resulting pellets were washed with 500 µL ice-cold acetone and centrifuged (18,000× *g* for 10 min at 4 °C). Pellets from each fraction were air-dried and kept at −80 °C until subsequent steps.

### 4.3. Proteomics Sample Preparation and Liquid Chromatography Coupled to MS/MS Analysis

Dried pellets from the DiffPOP fractionation were resuspended in 8 M urea/100 mM TEAB, pH 8.5. Proteins were reduced with 5 mM tris (2-carboxyethyl) phosphine hydrochloride (TCEP, Sigma-Aldrich, St. Louis, MO, USA) and alkylated with 50 mM chloroacetamide (Sigma-Aldrich). Proteins were digested overnight at 37 °C in 2 M urea/100 mM TEAB, pH 8.5, with trypsin (Promega) at an approximate ratio of 1:100 (enzyme/protein). Digestion was stopped with formic acid, 5% final concentration. Debris was removed by centrifugation. The final volume of each digest was 100 µL, and 5 µL of each digested fraction was used for analysis by liquid chromatography coupled to tandem mass spectrometry (LC-MS/MS).

The digested samples were analyzed on a Q Exactive mass spectrometer (Thermo Fisher Scientific, Waltham, MA, USA). The digests were injected directly onto a 2 cm desalting column attached to a 20 cm, 100 µm ID analytical column with a pulled tip. Both were packed with 3 µm Aqua C18 resin, (Phenomenex, Torrance, CA, USA, Cat. #04A-4311). Samples were separated at a flow rate of 400 nL/min on an Easy nLCII (Thermo Fisher Scientific). Buffer A was 5% acetonitrile and 0.1% formic acid; Buffer B was 80% acetonitrile and 0.1% formic acid. The following gradient was utilized: 1–35% B over 90 min, an increase to 80% B over 20 min, and held at 80% B for 10 min of washing for a 120 min total run time. The column was re-equilibrated with 10 µL of Buffer A prior to the injection of the sample. Peptides were eluted from the tip of the column and injected via nanospray directly into the mass spectrometer by application of 2.5 kV voltage at the back of the column. The Q Exactive was operated in a data-dependent mode. Full MS1 scans were collected in the Orbitrap at 70K resolution with a mass range of 400 to 1800 m/z and an AGC target of 1 × 10^6^. MS2 scans were acquired with a top 10 method, utilizing HCD fragmentation at 25 NCE, a resolution of 17.5K, an AGC target of 1 × 10^5^, and an underfill ratio of 0.1%. Maximum fill times were set to 60 ms and 120 ms for MS1 and MS2 scans, respectively. Quadrupole isolation at 2 m/z was used, singly charged and unassigned charge states were excluded, and dynamic exclusion was set to 15 s.

### 4.4. DiffPOP Data Processing for TargetSeeker-MS

The raw files were then analyzed with Integrated Proteomics Pipeline (IP2) and TargetSeeker-MS according to the previously published literature [30]. TargetSeeker-MS is a Bayesian inference-based algorithm that constructs a noise model for each protein using untreated (control) samples processed and quantified by MS, capturing variability across biological replicates. The algorithm then compares these control fractionation profiles to those of experimental cohorts processed under the same conditions. TargetSeeker-MS detects subtle, consistent changes in protein fractionation profiles across replicates, making it particularly effective for low-abundance proteins.

### 4.5. DiffPOP Data Processing with Skyline

Precursor ion intensities were extracted using Skyline (version 24.1.0.414) [62]. Charge 2 peptides GSPAINVAVHVFR (WT, *m*/*z* 683.8831), GSPAINVAMHVFR (V30M, m/z 699.8692), and GSPAINVAM(15.994815)HVFR (oxidation of M in V30M, m/z 707.8666) were used for quantitation.

### 4.6. Enzyme-Linked Immunosorbent Assay (ELISA)

ELISA assays were performed following the manufacturer’s protocol for the human C3a ELISA kit (Invitrogen, Vienna, Austria, Cat. #BMS2089) and the APOE (AD2) ELISA Kit (Thermo Scientific, Frederick, MD, USA, Cat. #EHAPOE).

### 4.7. SDS-PAGE and Immunoblotting

We utilized SDS-PAGE followed by immunoblotting with anti-C3 and anti-APOE antibodies to analyze samples from 3 healthy controls, 20 pre-symptomatic mutation carriers, and 20 patients from the previously reported Portuguese cohort before and after treatment (24 months) with tafamidis. These samples were chosen at random. The C3 antibody used (Invitrogen, Carlsbad, CA, USA, Cat. #PA521349) recognizes the N-terminus of the C3 α-chain (inactive form: 113 kDa).

### 4.8. Peptidomics Sample Preparation

Plasma (7.5 µL) was diluted in 142.5 µL of water, and proteins were precipitated by adding 2570 µL of 90% methanol/1% acetic acid. The sample was then vortexed and centrifuged (18,000× *g* for 10 min at 4 °C). The supernatant was collected, dried, and resuspended in 30 µL of 2 M urea and 125 mM Tris pH 8.5. The dissolved pellet was then loaded onto filter tips and analyzed with Q Exactive following the same protocol as mentioned above.

## 5. Conclusions

Our findings collectively open new avenues for research into the proteome alterations in amyloid diseases and elucidation of several key proteins that have the potential to be leveraged as biomarkers for V30M ATTRv-PN, including TTR itself, CP, APOE, and the complement pathway proteins such as C3. Although further investigation is required to deconvolute the relationship between inflammation (and its sequelae, like oxidative stress) and V30M ATTRv-PN pathogenesis—especially whether it causes or is caused by disease progression—this observation has direct disease relevance, as demonstrated previously by oxidative modification enhancing the amyloidogenicity of TTR [36]. Could inflammation and oxidative stress be the driving factors behind wild-type transthyretin polyneuropathy and/or cardiomyopathy? Our study provides valuable insights into the biochemical effects of tafamidis in ATTRv-PN patients’ plasma proteome, strengthening the evidence of its efficacy in mitigating ATTRv-PN disease. These findings not only enhance our understanding of ATTRv-PN pathogenesis but also open new avenues for therapeutic interventions and personalized treatment approaches in managing this debilitating disease.

## 6. Limitation of the Study and Future Directions

A limitation of this study is that the data were derived from a cohort defined by a single type of TTR mutation and restricted to a specific geographic region (Portugal), which may limit the generalizability of the findings to broader populations. Future studies could focus on validating these findings in larger experimental cohorts and developing predictive models based on these potential biomarkers to optimize treatment strategies. Additionally, investigating the long-term clinical outcomes associated with the observed proteome changes and other health metrics can guide future treatment decisions that can address the variability in response across patients. Finally, samples collected longitudinally for patients undergoing the conversion from asymptomatic to symptomatic disease were unavailable but would have been valuable for assessing our proposed biomarkers.

## Data Availability

All data available upon request.

## Abbreviations

The following abbreviations are used in this manuscript:

ATTR	Transthyretin Amyloidosis
ATTRv-PN	Transthyretin Amyloid Polyneuropathy
APOE	Apolipoprotein E
C3	Complement Component 3
C3a	Complement Component 3a
C3b	Complement Component 3b
C3c	Complement Component 3c
C5	Complement Component 5
C6	Complement Component 6
CP	Ceruloplasmin
LPA	Lipoprotein A
TTR	Transthyretin
V30M	Valine to methionine mutation at position 30 in TTR
